# Techno-economic assessment of hybrid extraction and distillation processes for furfural production from lignocellulosic biomass

**DOI:** 10.1186/s13068-017-0767-3

**Published:** 2017-03-29

**Authors:** Le Cao Nhien, Nguyen Van Duc Long, Sangyong Kim, Moonyong Lee

**Affiliations:** 1 School of Chemical Engineering, Yeungnam University, Gyeongsan, 712-749 South Korea; 2 Green Material and Process Group, Korea Institute of Industrial Technology, Cheonan, 31056 South Korea

**Keywords:** Biomass feedstock, Biorefinery development, Furfural, Hybrid process, Lignocellulosic biomass, Solvent selection

## Abstract

**Background:**

Lignocellulosic biomass is one of the most promising alternatives for replacing mineral resources to overcome global warming, which has become the most important environmental issue in recent years. Furfural was listed by the National Renewable Energy Laboratory as one of the top 30 potential chemicals arising from biomass. However, the current production of furfural is energy intensive and uses inefficient technology. Thus, a hybrid purification process that combines extraction and distillation to produce furfural from lignocellulosic biomass was considered and investigated in detail to improve the process efficiency. This effective hybrid process depends on the extracting solvent, which was selected based on a comprehensive procedure that ranged from solvent screening to complete process design.

**Results:**

Various solvents were first evaluated in terms of their extraction ability. Then, the most promising solvents were selected to study the separation feasibility. Eventually, processes that used the three best solvents (toluene, benzene, and butyl chloride) were designed and optimized in detail using Aspen Plus. Sustainability analysis was performed to evaluate these processes in terms of their energy requirements, total annual costs (TAC), and carbon dioxide (CO_2_) emissions. The results showed that butyl chloride was the most suitable solvent for the hybrid furfural process because it could save 44.7% of the TAC while reducing the CO_2_ emissions by 45.5% compared to the toluene process. In comparison with the traditional purification process using distillation, this suggested hybrid extraction/distillation process can save up to 19.2% of the TAC and reduce 58.3% total annual CO_2_ emissions. Furthermore, a sensitivity analysis of the feed composition and its effect on the performance of the proposed hybrid system was conducted.

**Conclusions:**

Butyl chloride was found to be the most suitable solvent for the hybrid extraction/distillation process of furfural production. The proposed hybrid sequence was more favorable than the traditional distillation process when the methanol fraction of the feed stream was <3% and more benefit could be obtained when that fraction decreased.

## Background

In recent years, the interest in renewable resources has increased considerably owing to environmental problems and the overdependence on mineral resources. Lignocellulosic biomass, which is the most abundant feedstock on the Earth, is a readily renewable resource for replacing fossil fuels. The use of this biomass feedstock for the manufacture of biochemicals and biofuels can gradually replace fossil-based feedstock. The conversion of biomass into chemicals conceptually brings a promise of sustainable, inherently safer, and eco-friendly production [1].

**Fig. 1:**
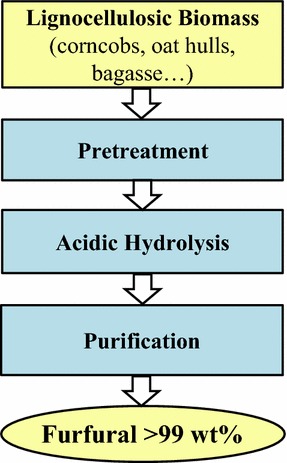
Schematic diagram of the furfural production from lignocellulosic biomass

Furfural, which is one of the top 30 potential chemicals arising from biomass, is a key bio-based platform chemical that can be used to replace oil-based chemicals [1]. Currently, its global production capacity is approximately 300 ktons/year, and it is primarily used for producing herbicides, stabilizers, pharmaceuticals, and numerous resins [2]. Zeitsh presented a comprehensive overview of traditional furfural technology and recent studies on this process [3]. Although the first industrial production of furfural was by the Quaker Oats Company in the early 1920s in Iowa, the current technologies used for furfural production have not been improved significantly [3]. Figure 1 shows a block flow diagram of the furfural production from lignocellulosic biomass. The typical furfural production process includes two main sections: reaction and purification. After pretreatment, a pentosane-rich biomass consisting of a material such as corn cobs or sugarcane bagasse is introduced to a series of reactors to be hydrolyzed to pentose, which is then dehydrated to furfural, using sulfuric acid as the catalyst. Subsequently, the vapor stream from the reactor, which consists of furfural (about 6 wt%), several byproducts (4 wt%), and water (making up the balance), is normally liquefied to make secondary steam before being purified using a distillation technique. In the reaction section, the low reactivity and poor mass transfer lead to a molar yield of furfural of about 50% in today’s process [3].

Meanwhile, in the purification part, removing a large quantity of water by distillation is very energy intensive because of the presence of a heterogeneous azeotrope between furfural and water (35.46 wt% furfural) [4, 5]. Note that this process is still used to produce approximately 80% of the furfural supply on today’s world market because of its low capital investment, easy implementation, and inexpensive raw materials [6]. The reaction and separation sections account for major portions of the total production costs. Therefore, improvements in these steps will lead to more cost-effective and sustainable processes.

In recent years, several processes have been reported in the literature and pilot-scale furfural production has been conducted, including the Supray Yield process, work of Vedernikovs, CIMV in France, Biorefinery by the Lignol Innovations Corporation in British Columbia, and multi-turbine column processes [3, 7–9]. Remarkably, these processes focused only on improving the furfural yield in the reaction part, without giving much attention to the energy-intensive purification part. A heat pump technique was applied in the pentose-to-furfural process to recover heat from the tops of the reactor and distillation column [10]. However, the use of a compressor might encounter several serious issues that could limit the use of a heat pump in the industry, such as the occurrence of overheating at a high operating temperature, high capital cost, and process complexity when operating the compressor equipment. Another investigation on the furfural purification process involved the use of a combined integrated and intensified technique, which showed a decrease in the total annual cost of about 10% [4]. Although distillation techniques require relatively high amounts of energy, they are still the most commonly used separation methods for purifying furfural on an industrial scale because of their ease of implementation and large capacity. It is worth noting that in the distillation process the presence of organic acids in the fluid at a high temperature will result in the loss of furfural [7].

On the other hand, liquid–liquid (LL) extraction may be more promising from the energy viewpoint. However, finding an effective solvent and designing a solvent regeneration part make the use of an extraction method more challenging. The solvent selected severely affects the performance of the extraction process. The solvent must be satisfactory in terms of its cost, selectivity, distribution coefficient, density, viscosity, toxicity, boiling point temperature, easy regeneration, etc.

Several methods for selecting extraction solvents have been well described in the literature, such as the tradition experimental method, computer-aided molecular design (CAMD) [11–15], and the combination of solvent screening and process design [16]. Carrying out multiple experiments to find a suitable solvent provides relatively accurate and reliable results, but there are clear limitations in terms of the time, cost, and a number of solvents tested. CAMD methods, on the other hand, can screen a large number of structural molecules and determine the molecule, which matches the target properties. In particular, a general CAMD can be formulated as a mixed-integer nonlinear programming (MINLP), in which several studies proposed the solution successfully [17, 18]. Tula et al. employed a CAMD approach to process design [11]. Note that if a solvent is selected without designing the entire process, including extraction and solvent regeneration units, it is not possible to evaluate the economic feasibility of the process. In contrast, a method that combines solvent screening and the design of the entire process can assess both the extraction abilities of the solvents and the economic feasibility and sustainability of the solvent processes [16].

In this study, hybrid extraction and distillation processes for furfural production from lignocellulosic biomass were designed and optimized through a comprehensive framework for solvent selection. Numerous solvents were initially screened on the basis of a literature review and preliminary simulations. Next, the separation feasibility of each potential solvent was considered before designing and optimizing the processes for the most promising solvents. All of the processes were simulated using Aspen Plus V9 and were assessed in terms of their energy requirement, economic performance, and environmental impact to make a fair comparison. Furthermore, the proposed hybrid process with the selected solvent was compared with the distillation purification process. In the lignocellulose-based process, feed composition uncertainties are inherent and may have an adverse effect on the suggested optimum. Therefore, a sensitivity analysis was also conducted.

## Methods

### Systematic procedure for solvent selection

**Fig. 2:**
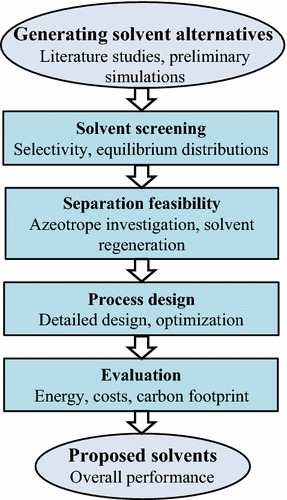
Systematic procedure for selecting solvent for furfural production

The selection of an effective solvent is critical to the design of the hybrid extraction/distillation process. The solvent should not only have a high equilibrium distribution and specific selectivity but also provide easy solvent regeneration and economic feasibility. In this study, the solvents were selected using a systematic procedure that was well described in our previous study [16]. As shown in Fig. 2, the comprehensive procedure for selecting a solvent for the production of furfural has essentially six steps. First, several preliminary simulations are carried out to select promising solvents from the literature. The selected solvents are then screened on the basis of their selectivity and equilibrium distribution. Next, azeotrope investigations and the ease of solvent regeneration are explored in the separation feasibility step. Processes using the most promising solvents are eventually designed and optimized in detail. The total annual costs (TAC) and carbon dioxide (CO_2_) emissions of all the processes are calculated to make a fair comparison, and the most promising solvents are accordingly proposed. The potential solvents and detailed designs for producing furfural on an industrial scale can be investigated using a framework.

### Simulation

Aspen Plus V9 was used to simulate the purification process for furfural production utilizing the RadFrac model for rigorous distillation and Extraction model for the LL extraction. The non-random two-liquid (NRTL) property method with binary parameters taken from the liquid–liquid equilibrium (LLE) Aspen database was used to calculate the liquid activity coefficients for the Extraction model. Meanwhile, the non-random two-liquid-Hayden–O’Connell (NRTL-HOC) thermodynamic package with binary parameters taken from the vapor–liquid equilibrium (VLE) Aspen database was used to perform a rigorous simulation of the distillation, in which the NRTL was used to calculate the liquid activity coefficients, and the HOC equation of state was used to calculate the thermodynamic properties of the vapor phase. The HOC equation reliably predicts a mixture of carboxylic acids, which may occur during the solvation of polar compounds and dimerization in the vapor phase [19]. Note that the interaction parameters between the major components, such as furfural–water and all solvents–water, are available in both the LLE and VLE Aspen databases. For the missing binary parameters of the remaining minor components, a Universal Quasichemical Functional-Group Activity Coefficients (UNIFAC) model was employed for estimation.

## Results and discussion

In this study, the furfural purification process was designed based on a production rate of 50 ktons/year. The feed stream consisted of aqueous solutions obtained from the acidic hydrolysis reaction of the lignocellulosic biomass. Table 1 lists the feed conditions, component compositions, and product specifications [3]. All of the solvents used have purities of more than 99 wt%. The solvent and feed streams are initially introduced to an LL extractor to produce a solvent-rich stream called the extract and an extracted-feed stream called the raffinate. The extract that contains the most furfural in the feed is input to a distillation column to purify the furfural and recover the solvent. The following sections show how different solvents were evaluated for the hybrid extraction/distillation process using the systematic framework shown in Fig. 2.

**Table 1:** Feed mixture conditions and product specifications

Component	Mass fraction (wt%)
Methanol	2.0
Water	90.0
Acetic acid (AA)	2.0
Furfural	6.0
Furfural product purity (wt%)	>99.0
Solvent product purity (wt%)	>99.0
Temperature (K)	353
Pressure (kPa)	101
Mass flowrate (kg/h)	105,000

### Solvent screening

**Fig. 3:**
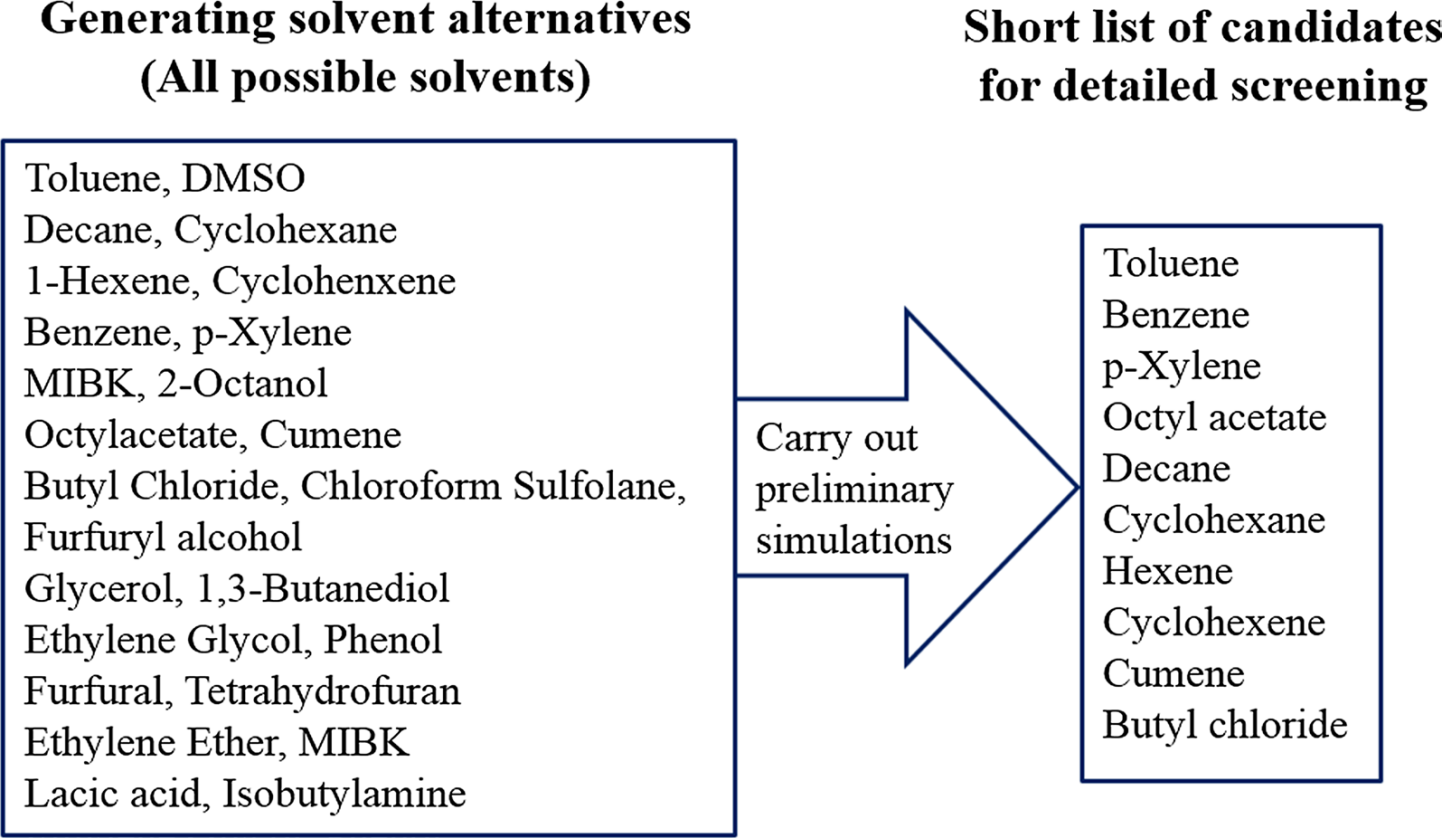
Lists of solvents: from possible initial solvents to short list of candidates for detailed screening

Because the art of solvent extraction has a long history, and LL extraction has been practiced in a large number of industrial applications, the key principles of solvent selection are available and well described in the literature [20–22]. Moreover, several researchers investigated the extraction of furfural from an aqueous stream using the acidic hydrolysis of biomass [4, 23]. On the basis of literature survey and heuristics, all possible solvents from a wide range of chemical families, including alcohols, normal hydrocarbons, ketones, aromatics, amines, etc., are firstly generated as shown in Fig. 3. Subsequently, several preliminary simulations are carried out in order to prepare a short list of candidates for detail screening step. In particular, ten potential solvents (toluene, benzene, *p*-xylene, octyl acetate, decane, cyclohexane, hexene, cyclohexene, cumene, and butyl chloride) were selected for further consideration.

The LL equilibrium curve plays a crucial role in designing an extraction process [24]. The use of equilibrium distribution coefficients that can screen a large number of solvents is an effective and economic method for solvent screening [25]. The distribution coefficient was calculated using the equilibrium data at a given temperature as follows [23]:

1 $$K_{\text{D}} = {{\left[ {\text{Extracted component}} \right]_{\text{solvent}} } \mathord{\left/ {\vphantom {{\left[ {\text{Extracted component}} \right]_{\text{solvent}} } {\left[ {\text{Extracted component}} \right]}}} \right. \kern-0pt} {\left[ {\text{Extracted component}} \right]}}_{\text{aqueous}}.$$

The distribution coefficient of the extracted component (*K*_D_) is the ratio of the weight percentage (wt%) of the extracted component [furfural, methanol, or acetic acid (AA)] in the solvent phase to the weight percentage of the extracted component in the aqueous phase. In the solvent screening step, the water–solvent equilibrium for furfural, AA, and methanol was studied, and the distribution coefficients and extraction efficiencies were compared for each solvent at 298 K. The feed flow rate was fixed, and the solvent flow rate was changed to analyze the solvent efficiency. In particular, for each solvent, four different cases with feed-to-solvent mass ratios of 1:0.8, 1:0.9, 1:1, and 1:1.1 were considered.

**Fig. 4:**
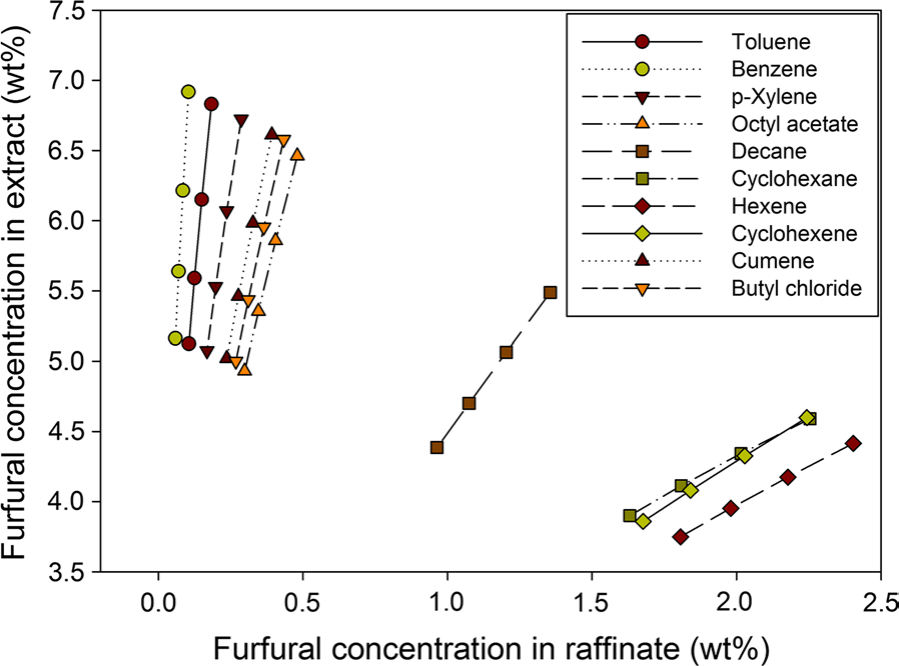
Equilibrium curves of furfural using different solvents at 298 K

Figure 4 shows the equilibrium curves of the furfural for the different solvents examined at 298 K. Clearly, benzene, toluene, *p*-xylene, cumene, octyl acetate, and butyl chloride have a favorable equilibrium with high distribution coefficient values, whereas decane, cyclohexane, cyclohexene, and hexane show unfavorable equilibrium for extracting furfural. Remarkably, benzene presents the most favorable equilibrium with the highest distribution coefficient value for furfural extraction. Accordingly, decane, cyclohexane, hexane, and cyclohexene were deemed unsuitable for extracting furfural from an aqueous solution.

**Fig. 5:**
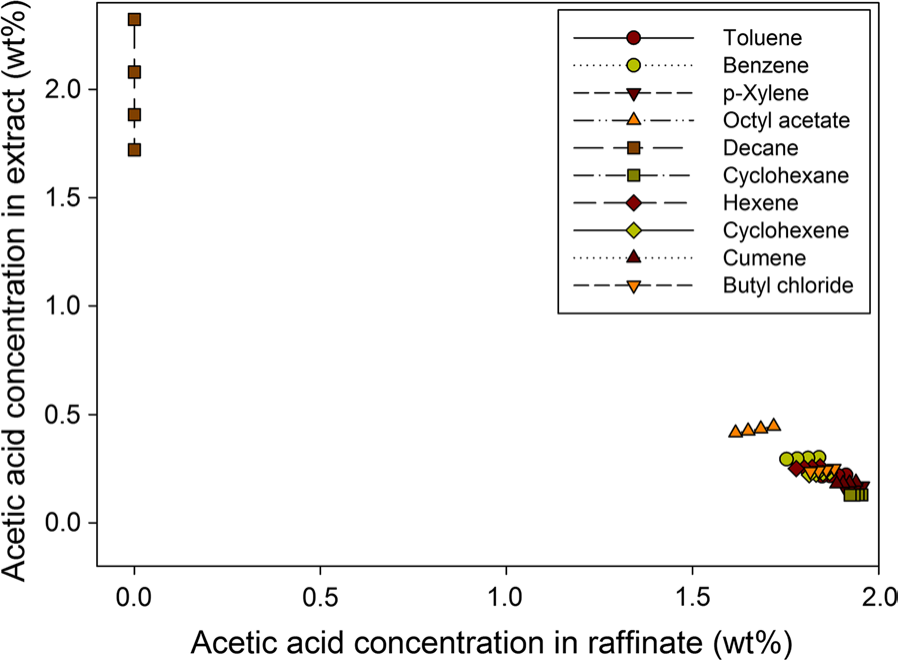
Equilibrium curves of acetic acid using different solvents at 298 K

Figure 5 presents the equilibrium curves of AA for all the solvents tested at 298 K. Interestingly, decane shows an excellent equilibrium with a very high AA concentration in the extract phase, whereas all of the remaining solvents have unfavorable equilibrium for AA extraction. In particular, with the exception of decane, the presence of AA in the raffinate phase was always much greater than that in the extract phase.

**Fig. 6:**
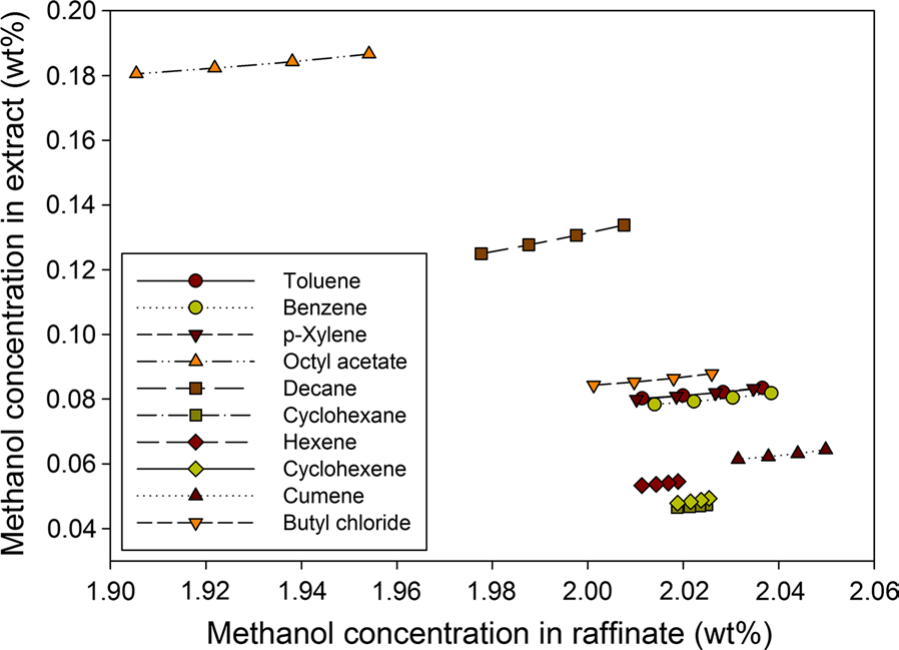
Equilibrium curves of methanol using different solvents at 298 K

Figure 6 shows the equilibrium curves of methanol for the different solvents examined at 298 K. Although octyl acetate shows the most favorable result for methanol extraction, the methanol concentration in the raffinate phase was much higher than that in the extract phase. As a result, all of the solvents tested were unsuitable for extracting methanol from the aqueous feed stream.

**Fig. 7:**
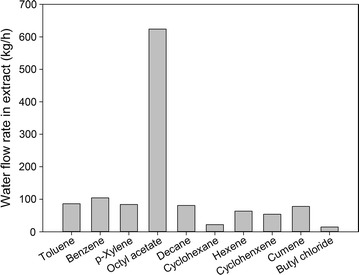
Water flow rate in extract for different solvents at feed-to-solvent ratio of 1:1

In addition to the extraction ability, the amount of water in the extract also has a great impact on the choice of solvent. If the extract contains a large quantity of water, greater effort for the subsequent distillation is needed to achieve the desired furfural purity, considering the heterogeneous azeotrope between the furfural and water. Figure 7 presents the amount of water in the extract for different solvents at a feed-to-solvent ratio of 1:1. The octyl acetate shows the highest water flow of about 600 kg/h, while the extracts of other solvents contain very small amounts of water. In the case of octyl acetate, because 600 kg/h accounts for only 0.6 wt% of water in the feed, it was still considered for the next step.

Based on the equilibrium data, the equilibrium distribution coefficients of furfural, AA, and methanol for all the solvents at 298 K were calculated as listed in Table 2. Cyclohexane, hexane, and cyclohexane, which had very low coefficients of furfural, were deemed unsuitable for furfural extraction compared to the other solvents. For AA extraction, only decane was promising, with an extremely high coefficient of AA. All of the solvents were deemed unsuitable for extracting methanol from the aqueous solution. It is worth noting that in the furfural production process, AA and methanol were treated as valuable byproducts. Hence, a solvent’s extraction ability for them has less impact than for furfural. In the case of decane, in addition to its excellent ability to extract AA, its performance in extracting furfural was still promising. Therefore, it was not eliminated in this step. Overall, benzene, toluene, *p*-xylene, cumene, octyl acetate, butyl chloride, and decane were selected for further consideration.

**Table 2:** Equilibrium distribution coefficient of furfural, AA, and methanol for different solvents at 298 K

Solvent	Feed-to-solvent ratio (mass)	*K*_D_		
		Furfural	AA	Methanol
Toluene	1:0.8	37.33	0.12	0.04
	1:0.9	41.11	0.12	0.04
	1:1	44.89	0.12	0.04
	1:1.1	48.68	0.12	0.04
Benzene	1:0.8	66.57	0.16	0.04
	1:0.9	73.76	0.16	0.04
	1:1	80.99	0.17	0.04
	1:1.1	88.25	0.17	0.04
*p*-Xylene	1:0.8	23.50	0.09	0.04
	1:0.9	25.73	0.09	0.04
	1:1	27.97	0.09	0.04
	1:1.1	30.22	0.09	0.04
Octyl acetate	1:0.8	13.46	0.26	0.10
	1:0.9	14.47	0.26	0.10
	1:1	15.50	0.26	0.09
	1:1.1	16.53	0.26	0.09
Decane	1:0.8	4.05	1E+07	0.10
	1:0.9	4.21	5E+07	0.10
	1:1	4.38	2E+08	0.09
	1:1.1	4.55	8E+08	0.09
Cyclohexane	1:0.8	2.04	0.07	0.02
	1:0.9	2.15	0.07	0.02
	1:1	2.27	0.07	0.02
	1:1.1	2.39	0.07	0.02
Hexene	1:0.8	1.84	0.14	0.03
	1:0.9	1.92	0.14	0.03
	1:1	2.00	0.14	0.03
	1:1.1	2.08	0.14	0.03
Cyclohexane	1:0.8	2.05	0.12	0.02
	1:0.9	2.13	0.12	0.02
	1:1	2.22	0.12	0.02
	1:1.1	2.30	0.12	0.02
Cumene	1:0.8	16.85	0.10	0.03
	1:0.9	18.31	0.10	0.03
	1:1	19.77	0.10	0.03
	1:1.1	21.24	0.10	0.03
Butyl chloride	1:0.8	15.20	0.13	0.04
	1:0.9	16.34	0.13	0.04
	1:1	17.50	0.13	0.04
	1:1.1	18.66	0.13	0.04

### Separation feasibility

If the added solvent forms azeotropes with the major extracted components, the design of the solvent regeneration part will become much more complex, leading to economic infeasibility. Therefore, in this step, the seven solvents selected in the solvent screening step were investigated to determine whether they formed azeotropes with the feed components to examine the ease of separation. Originally, furfural and water form a binary heterogeneous azeotrope (35.5 wt% furfural) at 371 K and 101 kPa. Table 3 lists all of the azeotropes formed between the added solvents and feed components, which were investigated based on the Aspen database at a pressure of 101 kPa. Because the major task of the hybrid extraction/distillation process is the separation of the furfural and solvent mixture, forming an azeotrope between the furfural and solvent will lead to either infeasible separation or economic infeasibility. Therefore, the *p*-xylene, decane, and cumene solvents were eliminated because they formed homogeneous azeotropes with furfural. Note that in all cases except octyl acetate, the extracts contained a very small amount of water (less than 100 kg/h). Thus, the effect of the heterogeneous azeotropes formed between the water and solvents was negligible. However, the extract of the octyl acetate process contained quite a large amount of water (600 kg/h when the feed/solvent mass ratio was 1:1). The fact that water not only forms a heterogeneous azeotrope with furfural but also with octyl acetate will make the following distillation process more complex and costly. Therefore, octyl acetate was excluded from further consideration. Overall, toluene, benzene, and butyl chloride were selected for the next process design step.

**Table 3:** Investigation of azeotropes between feed components and different solvents

Azeotrope	Solvent						
	Toluene	Benzene	*p*-Xylene	Octyl acetate	Decane	Cumene	Butyl chloride
Furfural-solvent	–	–	Homogeneous (97.1% *p*-xylene)	–	Homogeneous (45.5% decane)	Homogeneous (66.1% cumene)	–
Water–solvent	Heterogeneous (80.5% toluene)	Heterogeneous (91.1% benzene)	Heterogeneous (34.3% *p*-xylene)	Heterogeneous (16.4% octyl acetate)	Heterogeneous (42.7% decane)	Heterogeneous (57.8% cumene)	Heterogeneous (93.0% butyl chloride)
AA-solvent	Homogeneous (32.2% toluene)	–	Homogeneous (23.0% *p*-xylene)	–	Homogeneous (15.8% decane)	Homogeneous (4.3% cumene)	–
MeOH-solvent	Homogeneous (26.2% toluene)	Homogeneous (63.0% benzene)	–	–	–	–	Homogeneous (67.7% butyl chloride)

### Process design

In this step, the hybrid extraction/distillation processes for furfural production using the three best solvents (toluene, benzene, and butyl chloride) selected from the previous step were designed and optimized. The aqueous feed stream, which was cooled down to 313 K, and the solvent were first input to an extractor to produce an extract composed of a large portion of furfural in the feed and a raffinate. The extract was then introduced to a distillation column to separate the furfural and solvent. Note that in this hybrid process, the raffinate from the extractor consists of water loaded with AA and methanol which are delivered into a wastewater treatment plant. In particular, AA is not collected in both hybrid and traditional distillation processes while methanol is a by-product in the distillation process. Comparison of both processes will be discussed further in sustainability analysis section. For design specifications, all of the design variables such as a total number of trays, feed location, and feed-to-solvent ratio were manipulated through sensitivity analyses to improve the process efficiency while maintaining the product purities and recoveries. To make a fair comparison, the furfural recovery was 99.5 wt% through the extractor and 99.0 wt% through the distillation column, resulting in the same furfural production rate in all cases.

#### Toluene solvent process

**Fig. 8:**
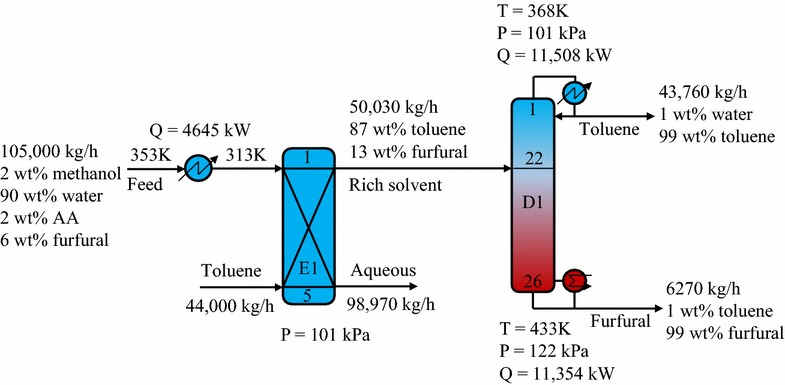
Schematic diagram of hybrid extraction/distillation process using toluene solvent

The feed stream obtained from the biomass hydrolysis process was first cooled down to 313 K before being introduced to the extractor (E1). Herein, the LL extraction process produced a raffinate at the bottom and an extract containing furfural at the top. The extract was then inputted to a distillation column (D1) to deliver a top stream of toluene and a bottom stream of the desired furfural. Figure 8 shows the key design and process parameters of the optimized hybrid extraction/distillation process using the toluene solvent. Because of the high furfural extraction ability, only 44,000 kg/h of toluene corresponding to a feed-to-solvent ratio of 1:0.4 was sufficient to extract 99.5 wt% of the furfural in the feed. The simulation results showed that D1 required medium-pressure steam for its reboiler and had an energy consumption of 11,354 kW.

#### Butyl chloride solvent process

**Fig. 9:**
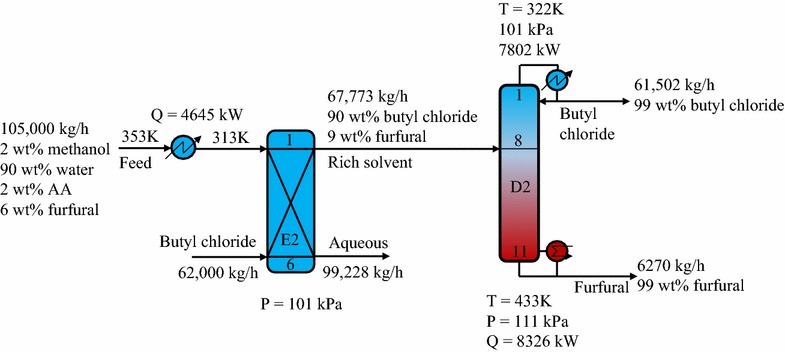
Schematic diagram of hybrid extraction/distillation process using butyl chloride solvent

Similar to the toluene process, the cooled feed stream and butyl chloride solvent in the butyl chloride process were input to an extractor to generate a solvent-rich phase and raffinate phase. The butyl chloride-rich stream was then introduced to a distillation column (D2) to isolate the furfural at the desired purity of 99 wt% and recover butyl chloride. Figure 9 presents the key design and process parameters of the optimized hybrid extraction/distillation process using the butyl chloride solvent. As shown in Fig. 4 in solvent screening section, butyl chloride showed a less favorable equilibrium for extracting furfural than toluene. Therefore, a greater amount of butyl chloride (62,000 kg/h) was needed to achieve a 99.5 wt% furfural recovery in the extractor. However, the separation in the distillation is based on differences in the mixture volatility. Thus, the separation of the butyl chloride–furfural mixture required less energy than the toluene–furfural separation. The results showed that the butyl chloride process can save 26.7% of the energy used by the toluene process.

#### Benzene solvent process

**Fig. 10:**
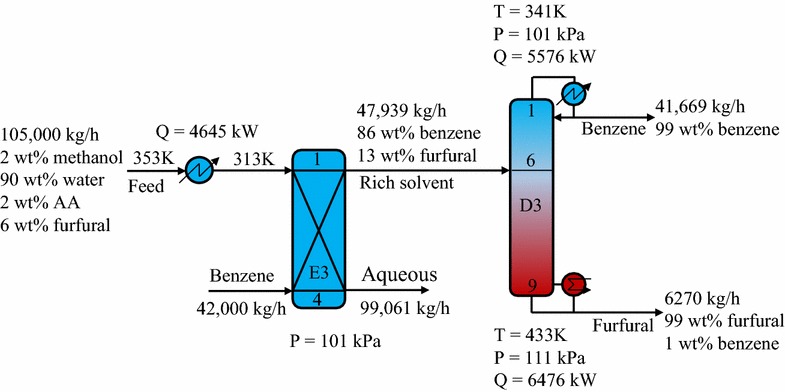
Schematic diagram of hybrid extraction/distillation process using benzene solvent

Figure 10 shows the simplified flowsheet and key process parameters of the optimized hybrid extraction/distillation process using the benzene solvent. The aqueous feed and solvent were introduced to an extractor (E3) to produce a benzene-rich phase at the top and raffinate phase at the bottom. Next, the benzene-rich stream was input to a distillation column (D3) to isolate the benzene and furfural at the desired purities. Among the solvents tested, benzene showed the highest distribution coefficient for furfural extraction. Hence, only 21,000 kg/h of benzene was needed to extract 99.5 wt% of the furfural in the feed, while the necessary amounts of butyl chloride and toluene were 62,000 and 44,000 kg/h, respectively. The results showed that the benzene process could achieve energy savings of 43.0 and 22.2% compared to the toluene and butyl chloride processes, respectively.

### Sustainability analysis

#### Economic and environmental evaluation

In an economic evaluation, the total investment cost (TIC), total annual operating cost (TOC), and TAC of all the processes were calculated based on the Turton textbook [26] as described in detail in our previous work [27]. The Chemical Engineering Index of 556.8 in 2015 was considered in this work. The column diameters, tray spacing, and column heights were calculated using the tray sizing function in Aspen Plus. The equipment considered in the investment costs consist of all of the reboilers, condensers, coolers, column vessels, and tray stacks. For the TOC calculation, the costs of a medium-pressure steam at 10 barg and cooling water are 14.19 and 0.354 $/GJ, respectively [26].

In addition, the total annual CO_2_ emissions (TCE) were also estimated to assess the environmental impact. For the steam reboilers, Gadalla’s modular method was applied to calculate the CO_2_ emissions [28]:

2 $$\left[ {{\text{CO}}_{ 2} } \right]_{\text{emiss}} = \left( {\frac{{Q_\text{{fuel}} }}{\text{NHV}}} \right)\left( {\frac{{{\text{C}}\% }}{100}} \right)\alpha,$$

where NHV is the net heating value of the fuel, and C% is the carbon content. For natural gas, the NHV is 48,900 kJ/kg, and C% is 0.41 kg/kg. The molar mass ratio of CO_2_ and C was *α* = 3.67. In addition, *Q*_fuel_ denotes the amount of fuel used, which was calculated as follows:

3 $$Q_{\text{fuel}} = \left( {\frac{{Q_{\text{proc}} }}{{\lambda_{\text{proc}} }}} \right)\left( {h_{\text{proc}} - 419} \right)\left( {\frac{{T_{\text{FTB}} - T_{0} }}{{T_{\text{FTB}} - T_{\text{stack}} }}} \right),$$

where *Q*_proc_ is the heat duty requirement of the system, and *λ*_proc_ (kJ/kg) and *h*_proc_ (kJ/kg) are the latent heat and enthalpy of the steam, respectively. The flame, stack, and ambient temperatures were *T*_FTB_ (2073 K), *T*_stack_ (433 K), and *T*_0_ (298 K), respectively.

Table 4 lists the key results for the carbon footprint and economic performance, including TAC, TIC, and TOC. Remarkably, the benzene process showed the best performance, achieving savings of 24.1, 60.7, and 57.1% in terms of TIC, TOC, and TAC, respectively, as well as a reduction of 43.0% in the carbon footprint compared to the toluene process. However, because benzene is a human carcinogen, its use as a solvent in the industry needs to be carefully controlled. In recent years, it has been supplanted by other safer solvents. Therefore, we here propose butyl chloride solvent as a good choice for the hybrid purification process of furfural because its process can save 17.1, 26.8, 25.9, and 26.7% in terms of TIC, TOC, TAC, and TCE, respectively, compared to the toluene process. Toxicity and eco-toxicity are main undesirable features of butyl chloride as a solvent. But if we look at its potential contribution to a global warming aspect, its significant CO_2_ emission reduction effect would give butyl chloride most attractive and advantageous feature as a solvent. As shown in the present study, the hybrid extraction and distillation process using butyl chloride solvent can reduce CO_2_ emission up to 58.3% equivalently compared to the traditional distillation process. Its outstanding performances on CO_2_ reduction due to enhanced energy efficiency with the high recovery of 99.71 wt% can be a worth consideration as a possible option of industrial furfural separation technology. Of course, to take into account its potential problem associated with the toxicity, it should be assumed that the process must be designed and operated to satisfy more strict safety and sealing regulation like as other processes handling harmful species.

**Table 4:** Comparison of key results for different solvent processes

Properties	Solvent		
	Benzene	Toluene	Butyl chloride
Condenser duty (kW)	5576	11,508	6995
Energy requirement saving in condenser (%)	51.5	–	39.2
Reboiler duty (kW)	6476	11,354	8326
Energy requirement saving in reboiler (%)	43.0	–	26.7
Investment cost (k$/year)	2761	3640	3018
Investment cost saving (%)	24.1	–	17.1
Annual operating cost (k$/year)	1969	5004	3663
Annual operating cost saving (%)	60.7	–	26.8
Total annual cost (k$/year)	2380	5547	4113
Total annual cost saving (%)	57.1	–	25.9
Total annual CO_2_ emission (ton/year)	13,923	24,411	17,901
Total annual CO_2_ reduction (%)	43.0	–	26.7

#### Comparison between hybrid extraction/distillation and tradition distillation processes

This section discusses how the proposed hybrid extraction/distillation process was compared with a traditional distillation process, which was presented in detail in our previous study [4], using the TAC to fully assess its economic feasibility. Table 5 lists the key results for the carbon footprint and costs for the two processes. Note that in comparison with the traditional distillation process, the hybrid process requires an added solvent and methanol is not collected as a byproduct. Therefore, additional costs for the extraction solvent and the benefit achieved from selling methanol were considered. To perform a fair comparison, the same production rate of 50 ktons of furfural per year was assumed for both processes and the relative annual costs (TAC*) were calculated from the original TAC. In the distillation process, the annualized methanol profit was subtracted from the original TAC, while in the hybrid process, the annualized cost of solvent makeup and the initial solvent cost were added to TAC and TIC, respectively. The prices of methanol and butyl chloride taken from global world market data were $0.276/kg [29] and $1.1/kg, respectively [30]. Meanwhile, the profit from selling methanol was assumed to be 50% of the total sales value.

**Table 5:** Comparison of key results between the hybrid and traditional distillation processes and their improved processes

Process	TCE (ton/year)	TIC (k$/year)	TOC (k$/year)	TAC (k$/year)	Methanol profit (k$/year)	Solvent cost (k$/year)	TAC* (k$/year)
Distillation process [4]	42,903	4501	8666	9337	−2306		7031
Hybrid process	17,901	3085	3663	4123		1558	5680
Savings (%)	58.3	31.5	57.7	55.8			19.2
Advanced distillation process [4]	37,938	4928	7661	8396	−2306		6089
Enhanced hybrid process	16,978	2935	3510	3947		1558	5505
Savings (%)	55.2	40.4	54.2	53.0			9.6

The results showed that the hybrid extraction/distillation process using the butyl chloride solvent can produce TAC savings of up to 19.2% compared to the traditional distillation process. Remarkably, the hybrid process was also more eco-friendly, accounting for a 58.3% reduction in the carbon footprint, which is closely linked to energy requirements, compared to the distillation process.

Furthermore, the results shown in Table 5 also indicated that the improved hybrid process, which utilizes the heat of the distillation bottom stream to preheat its feed, can save up to 9.6% of the TAC as compared to the improved distillation process using integrated and intensified techniques, which was studied by Nhien et al. [4].

#### Sensitivity analysis

In a lignocellulose-based process, breaking down the complex cellulose–hemicellulose–lignin structure of the lignocellulosic biomass could lead to an inherent uncertainty in the stream composition, which could have a major effect on the suggested design. Therefore, a sensitivity analysis was performed to improve the robustness of the proposed results. The aqueous feed of the present purification process produced from furfural reactors comprises about 6 wt% furfural, 90 wt% water, and 4 wt% byproducts (methanol and AA). In this analysis, the furfural and water fractions were fixed, and five scenarios for the methanol and AA composition of the feed were explored to determine how the byproduct composition affects the proposed design for the furfural purification process. Table 6 lists the key results of the sensitivity analysis of the hybrid and distillation processes.

**Table 6:** Sensitivity analysis of methanol and AA composition in the feed

Methanol–AA fraction (wt%)	0–4	1–3	2–2	3–1	4–0
Hybrid process					
Solvent makeup (ton/year)	1240	1320	1416	1568	1880
TAC* (k$/year)	5487	5575	5680	5848	6191
Distillation process					
Methanol product rate (ton/year)	0	8356	16,712	25,068	33,424
TAC* (k$/year)	8778	7913	7031	6150	5267

**Fig. 11:**
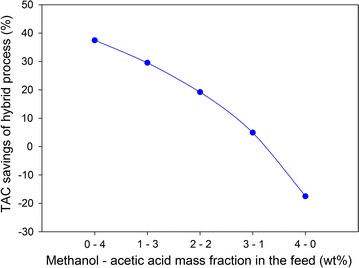
Effect of methanol–acetic acid composition on TAC savings of hybrid process

In the hybrid process, as the methanol fraction is increased, the rate of solvent makeup and methanol loss also increase. As a result, the TAC* gradually increases from $5.4 million up to $6.2 million with respect to a methanol fraction increase from 0 to 4 wt%. Note that changes in the byproduct composition did not have a significant effect on the hybrid design. In contrast to the hybrid process, as the methanol fraction increased, the TAC* of distillation decreased significantly. This means that the additional investment and operating costs for the methanol separation column due to an increase in the methanol flowrate are much lower than the profit from selling methanol. Figure 11 illustrates the benefit of the hybrid process compared to the distillation process with different scenarios for the byproduct composition. Remarkably, the hybrid process is more favorable than the distillation process when the methanol fraction of the feed stream decreases and does not produce any benefit. However, the distillation process is a better choice when the methanol fraction is larger than 3 wt%. It is worth noting that the present study used the UNIFAC model to estimate the missing binary parameters of several minor components. The experimental validation is essential to be considered as a next step for confirming process performance, leading to more reliable results for real implementation.

## Conclusions

A hybrid extraction/distillation process was proposed to successfully improve the traditional process of furfural production, which is energy intensive. Promising solvents were suggested through a comprehensive procedure for solvent selection that ranged from solvent screening to complete process design. The economic and environmental performances of three processes using the most promising solvents (toluene, benzene, and butyl chloride) were evaluated for a fair comparison. Overall, benzene and butyl chloride were found to be the most suitable solvents for furfural production because their processes could achieve TAC savings of 71.3 and 44.7%, respectively, compared to the toluene process. However, because of benzene’s obvious toxicity, butyl chloride was proposed as a good choice for the hybrid purification process of furfural. This suggested hybrid extraction/distillation process can save up to 19.2% of the TAC compared to the traditional distillation process. Furthermore, the hybrid process was also more eco-friendly, accounting for a carbon footprint reduction of 58.3% compared to the distillation process. Interestingly, the proposed sequence was more favorable than the distillation process when the methanol fraction of the feed stream was <3% and more benefit could be obtained when that fraction decreased.

## Abbreviations

CO_2_: carbon dioxide
CAMD: computer-aided molecular design
NRTL: non-random two-liquid
HOC: Hayden–O’Connell
VLE: vapor–liquid equilibrium
LLE: liquid–liquid equilibrium
LL: liquid–liquid
AA: acetic acid
NHV: net heating value
TIC: total investment cost
TOC: total annual operating cost
TCE: total annual CO_2_ emissions
TAC: total annual cost
TAC*: final total annual cost
UNIFAC: Universal Quasichemical Functional-Group Activity Coefficients
